# Development and Validation of a Resilience Skills Questionnaire for Health Sector Professionals Based on Social Cognitive Theory

**DOI:** 10.1155/2024/5660620

**Published:** 2024-01-06

**Authors:** Maryam Akbari, Hamidreza Mokarami, Rosanna Cousins, Changiz Rahimi Taghanaki, Mohammad Hossein Kaveh, Mehdi Jahangiri

**Affiliations:** ^1^Department of Health Education and Promotion, School of Health, Shiraz University of Medical Sciences, PO. Box 7536-75541, Shiraz, Iran; ^2^Department of Ergonomics, School of Health, Shiraz University of Medical Sciences, Shiraz, Iran; ^3^Department of Psychology, Liverpool Hope University, Liverpool, UK; ^4^Department of Clinical Psychology, School of Educational Sciences and Psychology, Shiraz University, Shiraz, Iran; ^5^Department of Occupational Health, School of Health, Shiraz University of Medical Sciences, Shiraz, Iran

## Abstract

Good resilience skills support effective and timely adjustment to demanding situations in the workplace. Existing tools are insufficient to develop and evaluate workplace interventions to improve employee's resilience skills. The aim of this study was to develop and validate a Resilience Skills Questionnaire (RSQ) using the key constructs of social cognitive theory—self-efficacy, self-regulation, and social support—as a theoretical framework. Following DeVellis' guidelines for scale development, first an expert panel of thirteen professors was recruited to support the item development stages and determine content validity. At this stage, the initial pool of 38 items was reduced to 25 items and CVR and CVI were calculated as 0.92 and 0.93, respectively, indicating good content validity. A second panel of ten health professionals confirmed face validity. An online survey comprised of the 25 developed items was then completed by 336 health professionals working in urban healthcare centers in Shiraz, Iran, in November 2021. The data were used to assess the psychometrics of the questionnaire according to its hypothesized three-dimensional structure. Confirmatory factor analysis yielded a final model of seventeen items in three dimensions, self-efficacy (six items), social support (six items), and self-regulation (five items), with good psychometric properties (*χ*^2^/df = 2.44 (*p* < 0.001), RMSEA = 0.06, GFI = 0.92, AGFI = 0.90, IFI = 0.93, CFI = 0.93). All standardized factor loadings were significant (*p* < 0.001). Internal consistency, as measured by Cronbach's alpha, was very good: RSQ (0.90), self-efficacy (0.86), social support (0.83), and self-regulation (0.86). Based on these results, the RSQ can be used as a standard and valid measure to develop and evaluate the effect of educational intervention programs to improve resilience skills and reduce job stress.

## 1. Introduction

Important strides have been made in the understanding of causes of work stress [1] and in developing risk assessment methodologies to ameliorate the psychological harm associated poor work design, organization, management, and social contexts [2, 3]. Nevertheless, the prevalence of stress, anxiety, and depression in the workforce remains high, and it has become clear that while risk assessment approaches are absolutely necessary [1–3], they are not sufficient to eliminate stress-related sickness absence and presenteeism. Critically, there are external factors and individual differences involved in mental health outcomes [4]. That is, resilience to positively adapt and successfully cope with necessary work demands is another important part of good mental health in the working population.

Resilience, as a construct, has its roots in historical investigations of children exposed to stressful life circumstances to identify and understand protective factors for these at-risk populations [5]. Based on their substantial study of adolescent adjustment to challenging life events, Masten et al. argued that resilience is a dynamic process and not a personality trait and—noting differences in the literature regarding the conceptualization of resilience—that the term resilience should be confined to representing consistent positive adjustment under challenging life conditions [6]. Working conditions, such as those seen for healthcare workers during the COVID-19 pandemic, can certainly fall into the category of challenging life conditions [7], and prevalence figures indicate that work-related mental health problems among a wide spectrum of employees are high [8]. If resilience can be conceptualized as having the insight and capability to cope in conditions of high risk or adversity, then educational interventions that can strengthen an employee's resilience will allow them to maintain or recover good mental health while facing challenging working conditions. To date, there are no evidence-based recommendations to improve resilience in the workplace, even though there is some understanding of predictors of resilience [5, 9–11].

The interest in resilience training is not new. In 2015, drawing on a decade of research into employee well-being and performance, Robertson et al. [12] reviewed fourteen intervention studies which suggested that resilience training could provide a number of benefits for both individuals and organizations. Their critique of the research methodologies, however, led to their conclusion that “there is no definitive evidence for the most effective training content or format” (p.533), because evidence of the skills involved in employee resilience was poor. In fact, only three of the fourteen studies provided a definition of resilience in line with the measure they used for evaluating their intervention. Ultimately, an effective training program to develop employee resilience must be founded on resilience skills. This starts with using an appropriate Resilience Skills Questionnaire to ascertain improvements in resilience and the efficacy of the intervention program.

A subsequent review of the literature concerned with individual resilience in the workplace identified 27 measures used to measure resilience [13]. This review described various shortcomings in the tools used in intervention studies to measure resilience. Briefly, some provided no information relating to the validity or reliability of the resilience scale used; most tools were unidimensional measures that conceptualized resilience as a stable trait for the purpose of identifying resilient individuals to understand a particular outcome (e.g. burnout and job satisfaction); and the focus of the studies that used a multidimensional resilience measure was on positive outcomes associated with resilient individuals, and thus, they do not provide information about determinants of resilience [13].

The social cognitive theory (SCT) [14] has been nominated as a means of improving understanding of resilience in the workplace [15]. The SCT has previously been used to provide a framework to interpret human behaviors in occupational settings [16] based on its premise that learning can occur in a social context through a dynamic, reciprocal interaction of the biological, personal, and environmental factors that contribute to the manifestation of resilience [17–20]. The SCT proposes that behavior, behavior change, and maintenance of behavior change are all a function of beliefs that the person has the resources to perform the behavior. The SCT accepts that the upkeep of behaviors over time needs environmental reinforcement and personal self-regulation, incorporating the notion of reciprocal determinism. These components of reciprocal determinism are influenced by various constructs of SCT of which self-efficacy, self-regulation, observational learning, and reinforcement through social support have been the most frequently utilized to explain and alter behavior [21]. Self-efficacy includes a person's confidence in their ability to pursue resilient behaviors, and thus, self-efficacy plays a central role in changing behaviors. Self-regulation includes setting goals and creating plans to perform resilient behaviors. Social support includes an individual's perception of the availability of emotional, informational, evaluative, financial, or instrumental support if needed [14, 15].

It has been suggested that resilience is a skill that can be learned and that providing knowledge and coaching can help improve coping styles, even in frontline healthcare workers during the COVID-19 pandemic [22, 23]. Although not directly rebutting the findings, it has been pointed out that evidence to answer the question of whether resilience can be taught is weak, because there is a need for robust tools to evaluate employees' resilience skills [11, 24, 25]. If this suggestion is to be examined, then such a tool needs to be developed. To our knowledge, this study is the first to develop a measure of resilience skills. This is the first step to develop an intervention to improve resilience skills and reduce work-related stress and associated mental health problems. The literature indicates that the SCT can be used as a suitable framework in developing and designing a tool to measure key determinants of resilience, and in view of the ongoing mental health challenges of those working to manage the COVID-19 pandemic [26], the health sector provides a suitable dynamic setting to validating a new tool. Therefore, the present study is aimed at developing and validating a new questionnaire to measure determinants of resilience in health sector employees based on the SCT.

## 2. Methods

### 2.1. Design

The study used a sequential exploratory mixed methods design following the DeVellis' guidelines for scale development [27], and the ten general recommendations of the COnsensus Study for the Selection of health status Measurement INstruments (COSMIN) were applied to the study [28]. First, a qualitative phase identified resilience items based on SCT constructs using an analysis of relevant literature and an expert panel to guide the development of dimensions. Then, a quantitative phase tested the psychometric properties of the developed questionnaire.

### 2.2. Item Development and Participants

To develop a Resilience Skills Questionnaire, first, we clearly defined what we wanted to measure. The content of the scale would be evidence-based and guided by the SCT as this theory was nominated as a useful framework for understanding resilience skills [13]. Initially, 38 items for the questionnaire were drawn from the academic literature on resilience skills, features of existing resilience scales, theoretical discussions about resilience, and the opinion of the Expert Panel. As shown in Table 1, the sample items (translated from Persian to English) were aligned with the three SCT constructs associated with resilience: self-efficacy (14 items), self-regulation (14 items), and social support (10 items).

We recruited an expert panel of thirteen professors in health promotion, psychology, and public health to support the item development phase. They were a targeted convenience sample recruited from universities. All participants were volunteers, and no payment was made to any participant. All gave written informed consent, and all understood their right to withdraw at any time.

Participants for the remainder of the questionnaire development were health professionals working in urban healthcare centers in Shiraz, Iran, involved in the planning and monitoring of the frontline COVID-19 healthcare services, the planning and monitoring of new COVID-19 vaccination centers, and evaluating service delivery during the pandemic. Urban health centers in Iran are provided as public services by government systems and provide a holistic model for healthcare [29].

The study was advertised in the health centers in Shiraz for three weeks mid-2021. The inclusion criterion was having at least one year of postqualification experience in the current place of work for the main study. To maximize recruitment, we posed no exclusion criteria on the presumption that the study was relevant to all employed in this context. There are many recommendations regarding the number of participants required for adequate statistical power in confirmatory factor analysis [30], as well as software which can support determination of sufficiency by demonstrating the minimum sample size required for 80% power. This is generally considered the minimum sample size for drawing accurate conclusions and avoiding type II errors [31]. Using the recommending settings [31], a G^∗^Power calculation (power 0.80, effect size 0.15, and alpha 0.05) for the initial 38 predictor items yielded a minimum sample size of 209 participants.

Altogether, 400 employees expressed an interest in the study, and full information sheets were sent to them. A total of 346 health sector workers who met the inclusion criterion, as confirmed by providing their job tenure on the written informed consent form, joined the study. From this sample, we randomly selected ten healthcare workers who identified that they have at least five years of work experience to support the face validity phase in the development of the items for the tool. The remaining 336 completed the developing tool to measure the psychometric properties of the developed Resilience Skills Questionnaire (RSQ). Based in the discussion of sample sizes for CFA by Kyriazos [30] and the G^∗^Power calculation to provide a sample with a minimum of 80% power, our sample size of 336 participants was sufficient to proceed.

### 2.3. Procedure

A face-to-face meeting of the thirteen members of the expert panel was instrumental in determining the format for item measurement. That is, for all items, the direction would be positive and the scoring should use a 5-point Likert agreement scale, strongly disagree = 1, agree = 2, neutral = 3, agree = 4, and strongly agree = 5, and higher scores would indicate better resilience skill status in each of the three SCT constructs.

The expert panel met a second time to examine the content validity of the tool and to ensure that the developed questionnaire was parsimonious and included only functional items. The initial item reduction used content validity ratio (CVR) and content validity index (CVI). Each expert was asked to independently classify each the sample items according to whether it was unnecessary = 1, somewhat necessary = 2, or necessary = 3. The formula (Ne − *N*/2)/(*N*/2), where Ne is the number of panelists who stated that the item was necessary and *N* is the number of panelists, was used to compute CVR [32]. According to Lawshe's table, the acceptable numeric value for CVR is 0.54 for thirteen experts [32]. Items that did not meet this threshold were removed from the item pool of the developing questionnaire.

CVI was then calculated by asking the expert panel to rate the relevance, clarity, and simplicity of the remaining items based on 4-point Likert scales. (Experts determined the relevance of each item in their opinions where not relevant = 1, relatively relevant = 2, relevant = 3, or completely relevant = 4; the simplicity of each item where not simple = 1, relatively simple = 2, simple = 3, and very simple = 4; and the clarity of each item where unclear = 1, relatively clear = 2, clear = 3, and very clearly = 4.) CVI was calculated by adding the relevance, clarity, and simplicity rating scores from each expert for each item, which were then divided by the total number of experts. Polit and Beck suggested CVI scores above 0.79 as appropriate [33].

The next step was to evaluate the scale to ascertain face validity using a sample of the target population [34]. For this purpose, the ten experienced health workers were asked to rate the importance of the remaining items to provide an impact score for each item, using a 5-point Likert scale (not important = 1, slightly important = 2, moderately important = 3, important = 4, and very important = 5), and the following formula: impact score = frequency × importance (frequency = the number of people who gave the item a score of 4 or 5; importance = mean score for each item).

#### 2.3.1. Quantitative Steps

The data collection phase took place in November 2021. The 38 sample items for the new questionnaire, and the 20-item Resilience at Work (RAW) scale [35], were made available to the participants through an online platform. The RAW scale was included to examine convergent validity. These are two tools that measure a form of employee resilience and while their purpose is not the same, they should share enough of the underlying general factor associated with resilience to yield a moderate to high correlation (i.e., *r* ≥ 0.05) when given to the same population [36]. The link to the questionnaires was distributed through a central email. A total of 336 completed anonymous questionnaires were submitted. Data were analyzed using IBM SPSS 24 and AMOS 24 (USA, SPSS Inc.). The initial inspection of the data confirmed that there were no floor and ceiling effects and that the database had less than 3% missing values. As the missing values were completely at random, they were replaced by personal mean scores, on the basis that there were few, and these supply minimally biased valuations in questionnaire research [37]. Internal consistency of the questionnaire at this stage was assessed using interitem and item-total correlations. Items with very low interitem and item-total correlations (*r* < 0.30) which would not be beneficial to a questionnaire were deleted [34]. A subsample of the participants (*n* = 25) agreed to complete the RSQ again two weeks after they had submitted their questionnaires to provide a preliminary examination of the stability of the developing questionnaire. The test-retest reliability was assessed using two-way mixed intraclass correlations (ICC) with absolute agreement. An ICC of less than 0.40 is indicative of poor reliability, between 0.40 and 0.59 is considered fair, between 0.60 and 0.74 is good, and an ICC of 0.75 and above indicates excellent reliability [38]. These reliability analyses were undertaken before assessing construct validity to ensure all the items in each of the three dimensions had sufficient discriminatory power, as required for measuring the considered dimensions.

Exploratory factor analysis (EFA; *N* = 200) and confirmatory factor analysis (CFA; *N* = 336) were performed to extract scale factors. EFA was performed using maximum likelihood with Promax rotation as this oblique rotation method allows factors to correlate with each other. In line with good practice, two statistical tests were applied to the data to ascertain factorability of the data [39]. The Kaiser-Meyer-Olkin (KMO) test was used to ensure that the ratio of the sample size to the number of items was sufficient. This would be confirmed by a KMO values ≥ 0.6 [39]. Bartlett's test of sphericity was used to assess the appropriateness of the correlations between variables in the factor model. A significant value (*p* ≤ 0.5) indicates factorability [39].

The extent to which the initial questionnaire was compatible with the SCT was examined using confirmatory factor analysis (CFA) with maximum likelihood. This statistical method of determining the structural validity of the developed questionnaire was appropriate: CFA is a deductive test of hypothetical models, and this is not possible using other multivariate analyses [40]. In this study, all variables were analyzed simultaneously to examine whether the model was consistent with the data. There are no definitive fit standards for CFA, but there are accepted test norms [34, 41, 42] and for the indices used to test the CFA model fit in this study. These were the chi-square test (*χ*^2^/df), the root mean square error of approximation (RMSEA), the goodness-of-fit index (GFI), the adjusted goodness-of-fit index (AGFI), and the comparative fit index (CFI). For a good model fit, the *χ*^2^/df ratio should be low. There is no absolute standard, and sample size complicates the use of this measure beyond a description of goodness of fit; however, a *χ*^2^/df ratio between 2 and 3 is generally regarded as an acceptable or good data-model fit. There is general agreement that RMSEA values ≤ 0.05 can be considered a good fit, and those between 0.05 and 0.08 are an adequate fit. Values of GFI and AGFI range from zero to one with values higher than 0.9 and 0.85 usually interpreted as an acceptable fit. CFI is an incremental relative fit index. Values range from zero to one and higher values are preferred. CFI ≥ 0.95 are indicative of a good fit [38, 41] although some sources suggest values ≥ 0.90 are acceptable [41].

Following the CFA, analysis of reliability of the final Resilience Skills Questionnaire (RSQ) in terms of internal consistency was assessed using Cronbach's alpha and interitem and item-total correlations. Alpha coefficients ≥ 0.70 have been considered acceptable for most scales, although an alpha coefficient between 0.80 and 0.95 suggests that a scale has good psychometric quality [41].

## 3. Results

### 3.1. Item Development

The content validity assessments showed that 13 of the sample items did not meet the required CVR value (0.54), so these items were removed. These were four self-efficacy items (8, 9, 12, and 14), six self-regulation items (6, 8, 9, 12, 13, and 14), and three social support items (6, 9, and 10) (see Table 1). The mean CVR score of the remaining 25 items was 0.92 (minimum = 0.69 to maximum = 1). The mean CVI for the 25 items was 0.93 (minimum = 0.84 to maximum = 1). These values indicate excellent content validity for all items. The impact score of each item was greater than 1.5, thus all items had face validity and were suitable for further analysis in the quantitative phase of the study.

### 3.2. Quantitative Results

**Figure 1:**
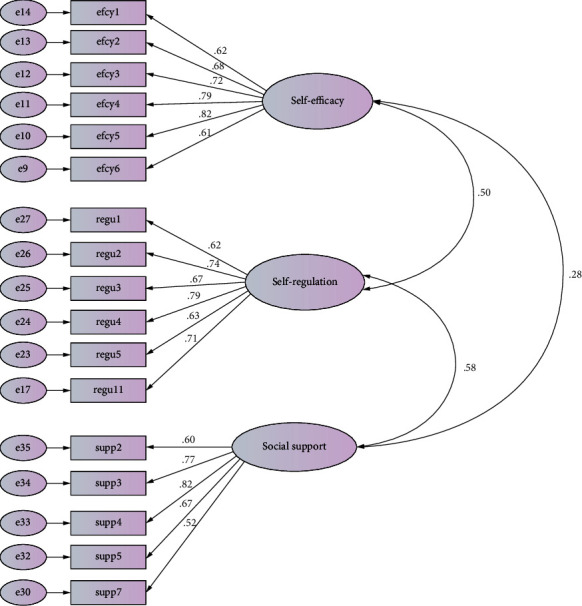
Confirmatory factor analysis with standardized item loadings according to dimension for the final 17-item, 3-dimension Resilience Skills Questionnaire. efcy = self-efficacy; regu = self-regulation; supp = social support.

A total of 336 health sector professionals completed the developed questionnaire. The mean age of the sample was 39.6 years (57.3% were female). The majority were married (74%). The samples were highly educated: 25.5% had a PhD, 38.8% had a master's degree, 32.5% had a bachelor's degree, and 3% had a high school diploma. We had confirmed the sufficiency of the sample size using G^∗^Power software, and even before the reduction of predictor items from 38 to 25, the sample size of 336 was sufficient to perform a CFA with the power which was set at the conventional 0.80. Construct validity of the three-dimensional model was examined by EFA followed by CFA. EFA of the data showed KMO = 0.89, and Bartlett's test of sphericity which was statistically significant indicated the factorability of correlation matrix. The findings of the initial model of the CFA indicated that the factor loadings of four items in self-efficacy construct (7, 10, 11, and 13), two items in the self-regulation construct (7, 10), and two items in the social support construct (1, 8) were lower than the cut-off point (*β* < 0.50), and the fit indices of the model were inappropriate. Thus, these items were removed (see final scale at the appendix), and a desirable model was achieved, which explained 53.4% of the total variance (as shown in Table 2 and Figure 1).

Construct validity for dimensions of the developed 17-item Resilience Skills Questionnaire (RSQ) was measured based on the CFA. The standardized regression weights were significant for all items in the final RSQ (*p* < 0.001). The loading factors of the RSQ items were significant (*p* < 0.001). The factor loadings were 0.63 for the 6-item self-efficacy dimension, 0.74 for the 6-item self-regulation dimension, and 0.65 for the 5-item social support dimension—all of which indicate high and favorable correlation between items and factor. In addition, all items of the questionnaire had acceptable internal consistency, and the relationships of each item with total score of each dimension were appropriate. The Cronbach's alpha coefficient of the RSQ was 0.903. Cronbach's alpha coefficients of each dimension and mean score (range 1-5), corrected item-total correlation, and Cronbach's alpha if item deleted of each dimension are presented in Table 3. The corrected item total correlations of the items in the three dimensions each had sufficient discriminatory power to measure the considered dimension. The table also presents an indication of excellent test-retest reliability (ICC) of the RSQ. Finally, Pearson's correlation analysis showed that RSQ scores were correlated with RAW scores which supported convergent validity of the RSQ (*r* = 0.588, *p* < 0.01). Items in the Resilience Skills Questionnaire according to subscales: self-efficacy, self-regulation, social support can be found in Table 4, which is presented as an appendix.

## 4. Discussion

The aim of this study was to develop and validate an instrument for assessing resilience skills based on the SCT to fill an identified gap in the literature [12, 13]. The objective was to provide a reliable and valid Resilience Skills Questionnaire to support the development of educational intervention programs to decrease the prevalence of work-related stress and other common mental health problems. We used a robust mixed method study design, informed by and compliant with the ten general recommendations of the COSMIN study design checklist for new measurement instruments [28]. This was designed to evaluate the methodological quality of research examining measurement properties [43]. Accordingly, the design and validation stages examined the content and face validity of the new instrument, including the metric properties of the considered items, the internal consistency of the SCT dimension items, and overall reliability and structural validity of the developed questionnaire. The final RSQ is comprised of 17 items and three theory-driven dimensions: self-efficacy (6 items), self-regulation (6 items), and social support (5 items).

To strengthen the validity of the new tool in its construction, we took advantage of the views of experts in psychology, health promotion, and public health to include different perspectives of resilience [27, 34]. The experts were involved in developing items and then in reducing the initial pool of potential items to include through assessing content validity. These steps contributed to ensuring that the final RSQ was parsimonious and made up of appropriate items [34]. The results confirmed that all the questionnaire constructs were suitable in terms of relevance, necessity, and clarity. The Cronbach's alpha for the whole scale and its dimensions also indicate that the developed RSQ has excellent internal consistency. Our initial test-retest result was excellent, and further research will confirm the degree of stability of results over time.

Previous studies have identified the potentials for resilience training to support employees and organizations to manage risks of work-related stress and improve performance [12, 13]. These studies also identified a gap in the availability of suitable robust scales to measure the skills that underpin resilience to support educational intervention programs. Pertinent to this study, the Hartman et al.'s review [13] included two scales developed to support further research in work-related performance. The 20-item Workplace Resilience Questionnaire [13] is a four-factor model that captures features of the resilient individual as a stable trait, and thus, this would be unsuitable as not in line with the assumption that resilience is a dynamic concept that can be improved. The RAW scale [35] was developed in Australia on the basis that resilience is a capability that can be developed to preserve the physical health of employees as well as improve engagement with work. RAW is a 20-item scale concerned with seven dimensions of workplace resilience; however, as suggested by the authors and from other studies, the RAW scale may have suboptimal reliability for two or three components which impacts on its usefulness [35, 44]. For example, a study to validate the scale for use in India [44] yielded only a partial replication, and the authors proceeded with a six-factor scale with 17 items. Also, another study in Australia reported a three-factor structure of the RAW [45]. Altogether, the RAW is insufficient for our overarching purpose of providing a tool to drive an intervention to improve employee's resilience skills to reduce the prevalence of work-related stress and other common mental health problems. This also supported our decision to take a theory-driven approach to developing a new scale based on SCT. Although the RAW scale has been criticized, it has been used as a valid measure of resilience, and hence, it was appropriate for examining convergent validity of the RSQ. This was shown.

Alongside the observation that there are various resilience scales measuring the concept from different perspectives [13], it has also been argued that there is a need for more robust evidence to address the fundamental question of whether resilience can be taught [11, 12]. With the development of the psychometrically sound RSQ, based on SCT, this theory-driven study has successfully approached the previous gap in the literature in terms of being able to develop interventions. The SCT was used to develop the new tool because it was conceptually appropriate and because previous research had particularly identified three component parts of the SCT as determinants of resilience [13, 15]. Future educational intervention studies using the RSQ will be able to test whether the general assumption that resilience can be taught is true and to what extent and in what contexts. The use of the RSQ developed in this study in intervention studies will serve to address these questions as well as support workplace wellbeing.

### 4.1. Strengths and Limitations

A strength of this study is that it has successfully developed a suitable Resilience Skills Questionnaire that was needed as a precursor to providing intervention programs to help employees to thrive in potentially stressful situations in the workplace [22]. It used a suitable sector to recruit participants in terms of the underpinning issues around managing work-related stress, particularly in 2021. There are many studies in the literature to confirm that the COVID-19 pandemic introduced an upward trend in the prevalence of mental health problems in the workforce, and a significant volume of these studies has referred to the health sector as being particularly as risk [46]. This provided the impetus for setting this study in the health sector and provides confidence that an appropriate population provided the data that underpinned the findings we report. Nevertheless, the RSQ was designed to be used across the range of work, and it will be necessary to test the psychometric properties in other types of workers. It follows from the latter point that a limitation of the present study was that data was collected from only one sector and one city in Iran due to financial constraints; therefore, evaluating the effectiveness of the RSQ in other studies is necessary. Another limitation is that the data are self-reported, and there is potential for social desirability bias to impact on some responses. An attempt to mitigate this bias was made through the use of anonymous online data collection.

## 5. Conclusion

This study developed and validated a questionnaire to measure determinants of resilience skills in health sector employees based on the SCT. The developed RSQ has appropriate psychometric properties, and it can be used as a standard and valid measure to evaluate the resilience skills of employees. This can, in turn, support longitudinal intervention programs to enhance employers' input into stress management and supporting the mental health of their workforce through the improvement of resilience skills. Future studies are required to confirm the generalizability of the RSQ in other areas of work.

## Figures and Tables

**Table 1:** Sample items for assessing resilience according to SCT constructs.

SCT construct	Sample items
Self-efficacy (efcy)	(1) When a problem upsets me a lot, I can evaluate my emotions, behavior, and beliefs about the problem. (2) If I hear my colleagues talking about me, I can still concentrate on my work. (3) If a colleague disrespects me, I can still consider the part I played in the incident. (4) When negative thoughts affect me, I can assess the pros and cons of those thoughts. (5) When I am very upset, I can reflect on the reason enough to approve or reject my negative thoughts. (6) When a problem makes me angry or distressed, I can think of ways to solve the problem. (7) Even when I am very busy, if unwanted thoughts come to me, I can dismiss them. (8) Even when I have a lot on my mind, I can listen to others when speaking with them. (9) While speaking with others, I wait for them to finish their point even if their conversation is too long or irrelevant. (10) Whenever I am talking with people, I can focus on their facial expressions. (11) If I am agitated by someone, I can plainly explain the reason to them. (12) I can comfort with my colleagues when a problem arises. (13) If I am asked to do something unusual, I can frankly decline to do it. (14) If I ever lose my temper with people, I can leave to room recognizing I need to calm down.

Self-regulation (regu)	(1) I evaluate my thoughts regularly. (2) When I evaluate my thoughts, I know that I should modify my thoughts. (3) I evaluate my relationship with others regularly. (4) When I evaluate my relationship with others, I consider how I can improve my relationships. (5) I evaluate my emotions regularly. (6) When I evaluate my emotions, I consider how I can modify what I do. (7) I have undertaken some training to help me understand my thoughts. (8) I have undertaken training or studied books to develop my communication skills. (9) I have undertaken resilience skills training to improve control of my emotions. (10) I always ask myself whether I could clear away my negative thoughts. (11) I ask my colleagues for feedback on whether I have improved my communications with others. (12) I evaluate my deeds regularly to know whether if I have been successful in controlling my emotions. (13) When I write down about my negative thoughts, I encourage myself. (14) When I manage to control any negative emotions, I encourage and appreciate myself.

Social support (supp)	(1) I have colleagues that help me with problems. (2) I have colleagues to motivate me when I am training to evaluate my thoughts. (3) My family help me to think about my communication skills. (4) I have at least one family member to guide me to know my emotions (e.g., anger, sadness, jealousy, anxiety, feeling blameworthy, and disappointment). (5) When I have negative emotions, I can talk about them with my family. (6) When I am upset or anxious, I can talk with my colleagues about it. (7) When I feel blameworthy for a mistake, I have friends to discuss them with me. (8) When I am under pressure at work, my family helps me with other issues. (9) When I am on leave, my colleagues help with my tasks. (10) If ever I need money, there are always some people who will help me.

**Table 2:** Fit indices of the confirmatory factor analysis of the RSQ.

Model fit index	Modified model
Chi-square/degrees of freedom (*χ*^2^/df)	283.444/116 = 2.44^∗∗^
Goodness-of-fit index	0.92
Adjusted goodness-of-fit index	0.90
Incremental fit index	0.93
Root mean square error of approximation	0.06

^∗∗^ *p* < 0.001.

**Table 3:** Mean (SD), corrected item-total correlations, Cronbach's alpha, *R*^2^, and ICC of RSQ.

Dimension	Item	Mean (SD)	Corrected item-total correlation	Cronbach's alpha if item deleted	Cronbach's alpha	*R* ^2^	ICC
Self-efficacy	Efcy1	3.37 (0.99)	0.607	0.843	0.859	0.384	0.869
	Efcy2	3.08 (1.0)	0.626	0.841		0.464	
	Efcy3	3.54 (0.95)	0.655	0.835		0.518	
	Efcy4	3.27 (0.97)	0.737	0.820		0.621	
	Efcy5	3.30 (0.94)	0.706	0.826		0.667	
	Efcy6	3.49 (0.99)	0.581	0.848		0.367	

Self-regulation	Sr1	3.31 (1.02)	0.576	0.857	0.863	0.385	0.823
	Sr2	3.54 (0.85)	0.715	0.831		0.542	
	Sr3	3.56 (0.85)	628	0.846		0.452	
	Sr4	3.17 (0.96)	0.741	0.825		0.625	
	Sr5	3.51 (0.84)	0.665	0.840		0.391	
	Sr11	3.04 (0.97)	0.643	0.843		0.498	

Social support	Ss2	3.41 (0.93)	0.537	0.814	0.825	0.359	0.887
	Ss3	3.57 (0.95)	0.689	0.769		596	
	Ss4	3.64 (0.90)	0.728	0.759		0.678	
	Ss5	3.57 (0.94)	0.607	0.794		0.447	
	Ss7	3.55 (0.85)	0.544	0.811		0.266	

**Table 4:** Items in the Resilience Skills Questionnaire according to subscales: self-efficacy, self-regulation, social support.

Statements
(1) When a problem upsets me a lot, I can evaluate my emotions, behavior, and beliefs about the problem. (2) If I hear my colleagues talking about me, I can still concentrate on my work. (3) If a colleague disrespects me, I can still consider the part I played in the incident. (4) When negative thoughts affect me, I can assess the pros and cons of those thoughts. (5) When I am very upset, I can reflect on the reason enough to approve or reject my negative thoughts. (6) When a problem makes me angry or distressed, I can think of ways to solve the problem.

(1) I evaluate my thoughts regularly. (2) When I evaluate my thoughts, I know that I should modify my thoughts. (3) I evaluate my relationship with others regularly. (4) When I evaluate my relationship with others, I consider how I can improve my relationships. (5) I evaluate my emotions regularly. (6) I ask my colleagues for feedback on whether I have improved my communications with others.

(1) I have colleagues to motivate me when I am training to evaluate my thoughts. (2) My family help me to think about my communication skills. (3) I have at least one family member to guide me to know my emotions (e.g., anger, sadness, jealousy, anxiety, feeling blameworthy, and disappointment). (4) When I have negative emotions, I can talk about them with my family. (5) When I feel blameworthy for a mistake, I have friends to discuss it with me.

1 = strongly disagree; 2 = agree; 3 = neutral; 4 = agree; 5 = strongly agree, Response.

## Data Availability

The data used to support the findings of this study are included within the article.

## Appendix

### The Resilience Skills Questionnaire

Instructions. Please indicate your agreement with the following statements from your experiences in your workplace using the following responses: 1 = strongly disagree; 2 = agree; 3 = neutral; 4 = agree; 5 = strongly agree.

## Abbreviations

RSQ: Resilience Skills Questionnaire
SCT: Social cognitive theory
CVR: Content validity ratio
CVI: Content validity index
CFA: Confirmatory factor analysis
*χ* ^2^/df: Chi-square/degrees of freedom ratio
GFI: Goodness-of-fit index
AGFI: Adjusted goodness-of-fit index
CFI: Comparative fit index
RMSEA: Root mean square error of approximation
COSMIN: COnsensus Study for the Selection of health status Measurement INstruments
RAW: Resilience at Work
ICC: Intraclass correlation
efcy: Self-efficacy
regu: Self-regulation
supp: Social support.
